# Direct and/or Indirect Roles for SUMO in Modulating Alpha-Synuclein Toxicity

**DOI:** 10.3390/biom5031697

**Published:** 2015-07-24

**Authors:** Shamini Vijayakumaran, Mathew B. Wong, Helma Antony, Dean L. Pountney

**Affiliations:** Menzies Health Institute Queensland, School of Medical Science, Griffith University, Gold Coast, Queensland 4222, Australia; E-Mails: shamini.vijayakumaran@griffithuni.edu.au (S.V.); mathew.wong@griffithuni.edu.au (M.B.W.); helmageorge@gmail.com (H.A.)

**Keywords:** alpha-synuclein, Parkinson’s disease, SUMO, multiple system atrophy, neurodegeneration, autophagy

## Abstract

α-Synuclein inclusion bodies are a pathological hallmark of several neurodegenerative diseases, including Parkinson’s disease, and contain aggregated α-synuclein and a variety of recruited factors, including protein chaperones, proteasome components, ubiquitin and the small ubiquitin-like modifier, SUMO-1. Cell culture and animal model studies suggest that misfolded, aggregated α-synuclein is actively translocated via the cytoskeletal system to a region of the cell where other factors that help to lessen the toxic effects can also be recruited. SUMO-1 covalently conjugates to various intracellular target proteins in a way analogous to ubiquitination to alter cellular distribution, function and metabolism and also plays an important role in a growing list of cellular pathways, including exosome secretion and apoptosis. Furthermore, SUMO-1 modified proteins have recently been linked to cell stress responses, such as oxidative stress response and heat shock response, with increased SUMOylation being neuroprotective in some cases. Several recent studies have linked SUMOylation to the ubiquitin-proteasome system, while other evidence implicates the lysosomal pathway. Other reports depict a direct mechanism whereby sumoylation reduced the aggregation tendency of α-synuclein, and reduced the toxicity. However, the precise role of SUMO-1 in neurodegeneration remains unclear. In this review, we explore the potential direct or indirect role(s) of SUMO-1 in the cellular response to misfolded α-synuclein in neurodegenerative disorders.

## 1. Introduction

### 1.1. Parkinson’s Disease

Parkinson’s disease (PD) is characterized by widespread intracellular inclusion bodies (Lewy bodies), composed largely of aggregates of the protein α-synuclein. Although the current evidence indicates that Lewy bodies are part of a protective cell response, namely the aggresome pathway, the cytotoxicity of abnormal soluble α-synuclein aggregates is implicated as a key factor causing cell death. The causes of α-synuclein aggregation are at present unclear; however, oxidative stress, post-translational modifications, calcium dyshomeostasis and proteolytic stress are key factors leading to the formation of cytotoxic α-synuclein species [1,2,3]. Indeed, intracellular α-synuclein aggregates are found in a variety of other neurodegenerative diseases, including multiple system atrophy and dementia with Lewy bodies, the so-called α-synucleinopathies, and are believed to form by a process involving the active translocation of soluble protein micro-aggregates along the microtubule network to converge on the microtubule organizing centre (MTOC/centrosome) [4,5,6,7]. Multiple modes of α-synuclein toxicity have been demonstrated, including membrane permeabilization by annular oligomers, binding to and inhibition of components of the 26S proteasome, inhibition of both macro- and micro-autophagy and the action of extracellular aggregates causing astrocyte and microglial activation [8,9,10]. The precise mechanisms are reviewed elsewhere [8,11]. Indeed, the sequestration of soluble cytotoxic α-synuclein species into filamentous aggresomes, although initially cytoprotective, might eventually also become toxic via the process of Lewy body maturation [12]. Moreover, reversal as well as prevention of α-synuclein aggregation may offer cytoprotection in α-synucleinopathy [13]. Aggresomes can be formed in neural cell culture under various conditions that result in the abnormal aggregation of cellular proteins and comprise amyloid-like α-synuclein filaments and a variety of recruited factors, including protein chaperones, proteasome components, ubiquitin, and recently, neuropathological inclusion bodies have been found to accumulate the small ubiquitin-like modifier-1, SUMO-1 [14,15]. Punctate SUMO-1-positive structures occurring as small (<1 µM) domains within pathological protein inclusions bodies and/or punctate SUMO-1 cytoplasmic staining in close proximity to inclusions is typical of the immunohistochemical distribution of SUMO-1 in a range of neurodegenerative disease pathologies. SUMO-1 has been found to be associated with both cytoplasmic inclusion bodies, such as Lewy bodies, in dementia with Lewy bodies and also with intranuclear inclusion bodies, such as polyglutamine aggregates, in Huntington’s disease and hereditary ataxias. Recent studies have suggested that there may be both direct and indirect links between SUMOylation and neurodegenerative disease pathology and the cell response to protein misfolding and aggregation. Thus, numerous aggregation-prone proteins linked to different diseases, including α-synuclein, have been shown to be SUMO substrates, suggesting a direct involvement in modulating protein solubility. Whereas, the identification of links between SUMO-1 and autophagy indicate that there may be indirect involvement in the cellular systems that tackle protein aggregate toxicity. In this review, we evaluate a range of factors that could provide links between SUMOylation and the toxicity of α-synuclein aggregates in neurodegeneration.

### 1.2. SUMO-1

SUMO, the Small Ubiquitin-like MOdifier, similar to ubiquitin, covalently conjugates to lysine residues in a wide range of substrate proteins, modulating the functional properties of the modified protein. Substrate proteins together with their binding properties can also be altered through the action of the SUMO pathway. There are three non-exclusive ways that this can be facilitated. SUMO modification can inhibit the binding site of a protein that interacts with the substrate protein by blocking the interaction site. Covalently attached SUMO could act as a hub for interaction by recruiting other binding partners. A conformational change in the SUMOylated substrate protein can modify its activity or expose previously marked binding sites [16,17]. As with ubiquitination, the mechanism of SUMOylation requires the coordinated actions of E1 (SUMO-activating), E2 (SUMO-specific conjugating, Ubc9) and, in most cases, E3 (SUMO ligase, e.g., PIAS3) enzymes that target recognition sequences within the target protein to covalently attach SUMO via C-terminal glycine to specific substrate lysine residues.

**Figure 1:**
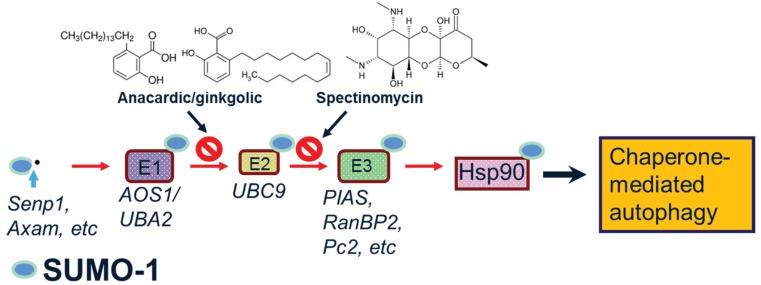
Steps in the SUMOylation of target proteins. SUMO-1 modification of substrate protein requires cleavage of the pro-protein and the action of E1, E2 and E3 enzymes. Specific inhibitors, ginkgolic acid/anacardic acid and spectinomycin can block E1 and E2 enzymes, respectively. SUMOylation of Hsp90 may modulate chaperone-mediated autophagy.

Reports have found supporting evidence that SUMO may be neuroprotective. Experiments proposed that overexpressing SUMO could increase cell survival under cellular stresses such as oxygen and glucose deprivation. Consequently, the knockdown of SUMO expression has proven deleterious, which provides strong evidence for a neuroprotective role for SUMO. Moreover, SUMO upregulation can protect against oxidative stress in ischemia-reperfusion. However, in diseases characterized by hyper SUMOylation of target proteins, down regulating SUMOylation of those proteins could be useful. Therefore, it is not surprising that the dysregulation of the SUMO pathway has been associated with neurodegenerative disorders [16,17,18]. The detailed biochemistry of SUMOylation has been discussed in several recent reviews [14,15,19,20,21,22]. Of the four mammalian SUMO isoforms identified, we will focus here on the SUMO-1 isoform that has been found associated with pathological inclusion bodies in several neurodegenerative diseases. Thus, unlike the SUMO-2/3 isoforms, SUMO-1 does not form polymeric SUMO-1 chains and does not have a large unconjugated cellular pool. The consequences of SUMOylation are context-specific, interacting across diverse intracellular pathways, including transcriptional regulation, mitochondrial fission and protein degradation, with recent studies implicating SUMO modification of the chaperone, Hsp90, in the protein refolding and autophagy-lysosome pathways [23,24]. Figure 1 illustrates the steps in SUMO-1 modification, illustrating Hsp90 as the target protein and specific inhibitors that can block the action of the E1 and E2 enzyme activities.

## 2. Potential Roles of SUMO-1 in α-Synuclein Aggregation, Degradation and Neuroprotection

### 2.1. SUMO-1 in α-Synuclein Disease

**Figure 2:**
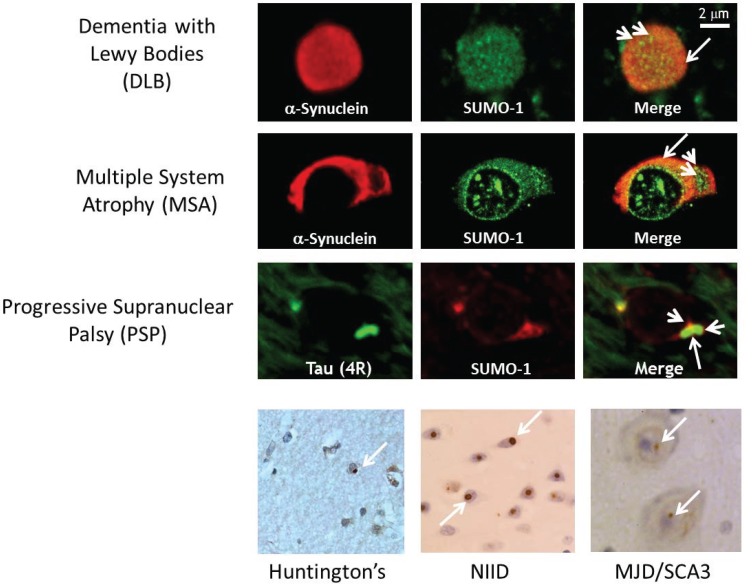
SUMO-1 is found in both intracytoplasmic and intranuclear protein inclusion bodies in neurodegenerative diseases. (**top**) Immunofluorescence of isolated Lewy bodies from dementia with Lewy bodies brain tissue (arrow) contain punctate SUMO-1 immunopositive domains (arrowheads). Multiple system atrophy glial cytoplasmic α-synuclein inclusion (GCI, arrow) body with associated nucleus shows SUMO-1 domains (arrowheads) surrounding the aggregated α-synuclein (For details see [26]). Progressive supranuclear palsy perinuclear glial tau inclusion bodies (large arrow) are decorated with SUMO-1 domains (arrowheads) (For details see [24]). (**Bottom**) Intranuclear neuronal inclusion bodies (arrows) in Huntington’s disease and spinocerebellar ataxias (NIID, MJD/SCA3) immunostain intensely for SUMO-1 (For details see [25]).

**Figure 3:**
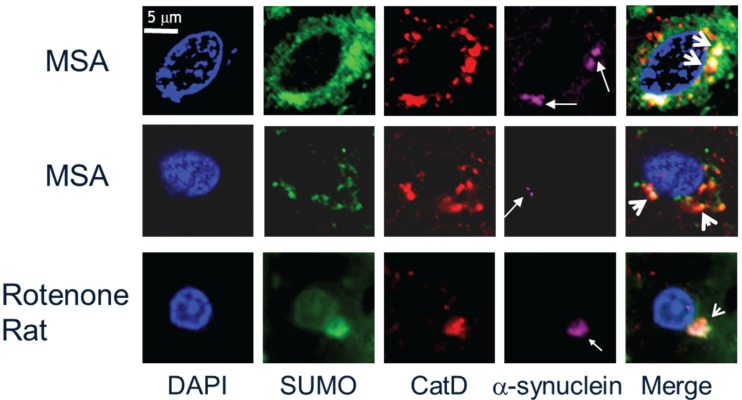
SUMO-1 marks lysosomes in α-synuclein inclusion body bearing cells in multiple system atrophy and rotenone rat α-synucleinopathy model tissue. Immunofluorescence triple labelling for SUMO-1, the lysosome marker, CatD, and α-synuclein reveals frequent colocalization of SUMO-1 with lysosomes (arrowheads) in close proximity to α-synuclein inclusion bodies (arrows) [24].

Central to the cytotoxicity of α-synuclein in disease pathogenesis is the accumulation of aggregated or oligomeric forms of the protein due to aberrant protein folding. The major pathological hallmark of PD is the presence of Lewy bodies with the major constituent being accumulated aggregated forms of the α-synuclein protein [4,6]. Thus, SUMOylation can impinge on the aetiology of α-synucleinopathy in one of two ways, either by modulating the propensity of α-synuclein to aggregate or by affecting the action of the cellular degradative machinery in clearance of α-synuclein aggregates, or indeed by a combination of both. SUMO-1 was first reported to be colocalised with pathological intranuclear protein aggregates in human neurodegenerative diseases [25]. Figure 2 (bottom panels) illustrates intranuclear inclusion bodies (white arrows) immunopositive for SUMO-1 (brown) in cortical tissue sections from Huntington’s disease, Machado Joseph disease/spinocerebellar ataxia type-3 (MJD/SCA3) and neuronal intranuclear inclusion disease (NIID). SUMO-1 was then found to be associated with α-synuclein immunopositive inclusion bodies in cases of multiple system atrophy and dementia with Lewy bodies, where small subdomains within the inclusion body structures were immunolabelled for SUMO-1 [26]. Figure 2 (top panels) shows the characteristic punctate features (arrowheads) with SUMO-1 immunofluorescence (green) observed in the glial cytoplasmic α-synuclein inclusion bodies of multiple system atrophy and the cortical Lewy bodies of dementia with Lewy bodies. It is noteworthy that the SUMO-1 positive puncta are clustered in regions of the inclusion bodies with relatively little α-synuclein immunoreactivity, similarly to that observed for the glial tau inclusion bodies of progressive supranuclear palsy shown for comparison. SUMO labelling of Lewy bodies in Parkinson’s disease tissue was subsequently demonstrated by Kim and co-workers, who also showed that SUMO was recruited to α-synuclein inclusions induced by proteasome inhibition in cultured cos-7 cells [27]. Immunocapture studies then revealed that SUMO-1 was associated with several proteins (NSF, dynamin, Munc18 and Hsp90) in preparations of pathological inclusion bodies with mechanistic links to the endomembrane system [28]. More recently, SUMO-1 was found to be associated with lysosomes and Hsp90 in glial protein aggregate diseases, including the α-synucleinopathy, multiple system atrophy [24]. Thus, in human diseased tissue, SUMO-1 conjugated to Hsp90 was found to be associated with lysosomes or lysosome remnants clustered around or embedded in α-synuclein inclusion bodies, indicating a link to the autophagy response, rather than being directly conjugated to α-synuclein itself. Furthermore, the SUMOylation status was investigated in a unilateral oxidative stress mouse model of α-synuclein disease, whereby Western analysis of the brain homogenates showed statistically significant increases of both SUMO-1 and α-synuclein in the lesioned hemisphere compared to the un-lesioned hemisphere. Furthermore, SUMO was found to be associated with lysosomes clustered around α-synuclein intracellular inclusion bodies in both mouse and rat models and isolations of lysosomes from the mouse brain homogenates indicated increased SUMOylation of Hsp90 in the lesioned hemisphere as compared to the un-lesioned hemisphere [24,29]. Figure 3 illustrates the pronounced SUMO-1 immunofluorescence of lysosomes (arrowheads) marked by cathepsin D (CatD) in multiple system atrophy and rotenone rat α-synucleinopathy model tissue sections found associated with α-synuclein inclusion bodies (arrows). Moreover, an *in vitro* study investigated the direct effects of SUMOylation on the aggregation susceptibility of α-synuclein. The study revealed that SUMOylation of a small portion of α-synuclein was adequate to avoid its aggregation [30]. In agreement with these *in vitro* experiments, cell culture studies have revealed that SUMO deficient α-synuclein exacerbated aggregation, causing detrimental increases in cellular toxicity [31]. Taken together, these latter studies suggest a potential neuroprotective role for SUMO in maintaining the solubility of α-synuclein [32], although SUMOylation of α-synuclein by the human polycomb protein has also been found to promote inclusion body formation [33]. Besides modulating the propensity of α-synuclein to aggregate and clearance of α-synuclein aggregates so as to inhibit degeneration, the reversal of α-synuclein aggregation by upregulating molecular chaperones to restore protein homeostasis (proteostasis) may also impinge on α-synucleinopathy, as this strategy is able to reverse degeneration [10,13].

### 2.2. SUMO-1 in α-Synuclein Aggregate Clearance

Intracellular mechanisms for the clearance of aberrantly folded proteins include two proteolytic pathways: The autophagy-lysosome pathway and the ubiquitin-proteasome system (UPS), both of which have potential roles for the SUMO-1 modification. While autophagy is a “self-eating” mechanism essential for selective/non-selective protein turnover, often of long-lived proteins, the UPS facilitates selective and rapid proteolysis of short-lived proteins via ubiquitination. Together, these pathways aid stress adaptation and maintain proteostasis in the cell. In UPS, degradation is ubiquitin-dependent and occurs at the 26S proteasome, an organelle consisting of a barrel-shaped 20S proteolytic core capped at both ends by the 19S regulatory subunits. Ubiquitin is capable of forming polyubiquitin chains at seven different lysine residues: K-6, K-11, K-27, K-29, K-33, K-48 and K-63. Client proteins for proteasomal degradation are marked by “substrate-specific” ubiquitin E3 ligases, such as CHIP (carboxy terminus of Hsc70 interacting protein), Parkin, *etc.*, via covalent polyubiquitination linked through K-48 residues. These ubiquitin multimers are recognised by the polyubiquitin binding receptors (Rpn10/Rpn13) of the 19S regulatory particles and de-ubiquitinated. Following this, the ATPase component of the 19S mediates substrate unfolding, 20S gate opening and substrate channelling into the 20S core for proteolysis. Finally, the peptide bonds of the client proteins are hydrolysed by the β-type subunits of 20S that possess caspase-like, trypsin-like and chymotrypsin-like activitites. Multiple factors affect UPS degradation. The prime rate-limiting factor is the substrate unfolding step, which is indispensable for allowing entry into the narrow 20S chamber. Hence, it is possible for large protein aggregates of the neurodegenerative diseases that cannot unfold to block this pathway. Multiple shuttling factors that deliver polyubiquitinated proteins via multiple direct and indirect routes have also been observed. Most factors contain a “ubiquitin-like” domain (UBL/UBX) at the *N*-terminus that binds the 26S proteasome and a “ubiquitin-interacting motif” (UIM) or “ubiquitin-associated” domain (UBA) at the C-terminus that binds polyubiquitinated proteins e.g., p62/sqstm1 (sequestome 1) protein, Rad23, DSK2, *etc.* Alternatively, some ubiquitin E3 ligases, e.g., Parkin, CHIP and VHL (von Hippel Lindau), possess their own UBL domains, or interact with proteins that have UBL domains, for proteasome binding. In addition, some of these shuttling factors are reported to deliver proteins to VCP (valosin-containing protein)/p97/Cdc48 complexes for unfolding prior to their delivery to proteasomes [34,35,36,37,38].

**Figure 4:**
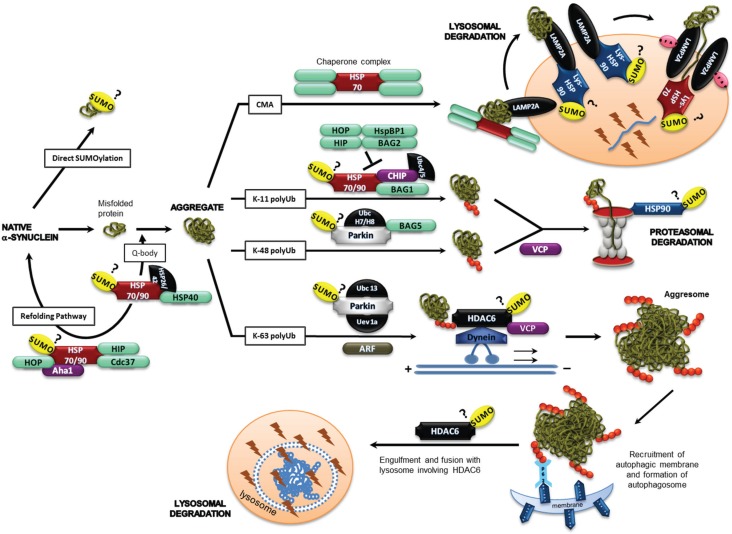
Mechanistic model for the role of SUMO-1 in protein misfolding. SUMO-1 post-translational modification of target proteins can modulate α-synuclein aggregation, refolding and aggregate clearance. SUMOylation of one or more of the potential substrate proteins illustrated in a variety of cellular pathways associated with protein aggregation (Hsp90, HDAC6, p97/VCP, TRAF6, ARF, Parkin, α-synuclein) could link SUMO-1 to the cellular response to aggregated α-synuclein in neurodegenerative disease. See text for details.

In autophagic proteolysis, recruitment of aggregated/misfolded proteins occurs into lysosomes with subsequent digestion in the lysosomal lumen. Depending on the mode of cargo delivery, autophagy has been differentiated to three sub-types: Microautophagy, macroautophagy and chaperone-mediated autophagy (CMA). In macroautophagy, cargo is enclosed by a double-membraned vesicle called the autophagosome that fuses with the lysosome for digestion, whereas, in microautophagy, cargo is directly engulfed by the invagination of lysosomal membrane. In CMA, cargo is actively transported into the lysosomal lumen for proteolysis after client proteins are recognized by the cytoplasmic chaperone (Hsc70/heat shock cognate70) [39]. Upon recognition, modulatory co-chaperones BAG1, HIP, HOP and Hsp40 along with Hsc70 target these proteins to the lysosomal surface. But lysosomal entry is permitted only to those substrates that are capable of complete unfolding, similar to the UPS process. Translocation across the lysosomal membrane into the lumen is facilitated by LAMP-2A (lysosome-associated membrane protein type 2A) and the luminal forms of Hsc70 (lys-Hsc70) and Hsp90 (lys-Hsp90) [39,40] and can be inhibited by α-synuclein mutants and aggregates [41] . Moreover, α-synuclein is also implicated in the inhibition of macroautphagy via interaction with rab1a [42,43]. Emerging evidence indicates that micro- and macroautophagy can also selectively target specific organelles or protein aggregates (aggrephagy) for degradation (reviewed in [38,44]). Surprisingly, ubiquitination is found to act as a recognition signal for selective autophagy as well (reviewed in [35,45]). In this context, it is noteworthy that the topology of ubiquitin linkage, particularly K-63-linked polyubiquitin, has significance in both inclusion body formation and/or subsequent clearance in several neurodegenerative disorders [46,47,48]. In selective autophagy of protein aggregates, *i.e.*, aggrephagy (see Figure 4), the key molecular players identified to date are p62/sqstm1, NBR1 (neighbour of Brca1 gene), LC3 (microtubule-associated protein 1 light chain 3) and HDAC6 (histone deacetylase 6). A multi-functional protein, p62, harbours multiple protein-protein interaction domains. Therefore, in addition to functioning as a shuttling factor of polyubiquitinated proteins for proteasomal degradation (as mentioned earlier), p62 also modulates formation of aggregates [49] and aggresomes [50] and promotes their autophagic clearance [48,49,50]. The autophagic degradation mediated by p62 is said to be selective due to its preferential binding of K-63-linked polyubiquitinated proteins [48] and recruitment of the autophagosomes via interacting with LC3 [51]. LC3 is an essential component of the autophagosomal membrane and is conjugated to phosphoethanolamine (PE) during the formation of autophagosomes [52]. Thus, p62 acts as an adaptor protein between aggregated protein and autophagosomes. The identification of other p62-like proteins, e.g., NBR1 [53] and Nix [54], has led to the recognition of a LC3-interacting (LIR) motif as being responsible for the specific recruitment of autophagosomes to protein aggregates [35,55]. HDAC6, a microtubule-associated deacetylase, is suggested to act downstream of p62 in this pathway [45]. Interestingly, HDAC6 is reported to act at two stages. First, it enhances aggresome formation via simultaneously binding K-63-linked polyubiquitinated proteins and dynein motors to retrogradely transport misfolded proteins along the microtubules to the aggresome [56,57]. Enhanced aggresome formation by HDAC6 has been observed in PD. Second, it controls the autophagosome-lysosome fusion by recruiting the required actin-remodelling machinery [58]. In fact, a deficiency of HDAC6 can result in protein aggregate build-up and neurodegeneration while the presence of HDAC6 can confer neuroprotection by facilitating autophagic degradation of toxic proteins [59,60].

SUMO-1 has been identified to specifically co-localize with lysosomes and in α-synuclein aggregate-bearing cells in a human glial cell culture model under proteasome inhibition [24]. Although the lysosomal and proteasomal proteolysis pathways were previously thought to function independently, recent evidence indicates the existence of specific mechanisms for collaborative functioning between selective autophagy pathways and proteasomal degradation, especially in the event of toxic accumulation of protein aggregates. This crosstalk is considered to be made possible by the dual role of ubiquitination which acts as the protein degradation signal for both UPS and the selective mode of macroautophagy [35,45]. However, the roles of other post-translational modifiers like acetylation, SUMO, *etc.* have been overlooked. Increased acetylation of a lysine residue in Htt has been reported to facilitate enhanced macroautophagic clearance and thereby reverse toxicity in cellular and transgenic models of HD [49,61]. Similarly, the SUMO post-translational modification is found in the inclusion bodies and reported to associate with neurodegenerative disease-specific proteins to produce substrate-specific effects. SUMO has also been observed to associate with and/or functionally modulate several key players of proteasomal as well as lysosomal pathways (discussed below). Furthermore, clustering of SUMO-1-positive lysosomes within and around pathological protein inclusion bodies in MSA and PSP has recently been observed. Figure 3 shows examples of lysosomes that are either embedded in or surround α-synuclein intracytoplasmic aggregates in MSA and rat model tissue, respectively. SUMO-1 was also found to be associated with lysosomes in cellular models of α-synucleinopathy, tauopathy and polyglutamine disease [24]. These findings suggest that SUMO may play a direct and/or indirect role in the cellular response to α-synuclein misfolding and aggregation in neurodegenerative diseases. In an attempt to define the molecular aspects of this mechanism, we have surveyed the existing literature for links to the potential roles of SUMO in the cell response to aggregated α-synuclein, summarized in Figure 4 and below.

### 2.3. α-Synuclein and SUMOylation

α-Synuclein is a 14kDa protein encoded by the SNCA gene that is highly conserved in vertebrate species. Although the exact role of α-synuclein remains unclear, the protein is primarily localised to presynaptic terminals of dopaminergic neurons [62] and is thought to be involved in neurotransmitter vesicle recycling and dopamine neurotransmission through interaction with soluble NSF attachment protein receptor (SNARE) [63]. α-Synuclein has been shown to interact with membranes [64] and function as a SNARE associated protein mediated by Rab3a [65]. Interestingly, Krumova *et al.* have shown recently that SUMOylation of α-synuclein *in vitro* results in a less aggregation-prone protein. In animal model studies, neuron-specific expression of the SUMO-2 isoform was shown to cause α-synuclein SUMOylation and mutation of lysine residues in the hydrophobic NAC domain that can be SUMOylated was found to reduce the toxicity of α-synuclein when over-expressed in rat brains from a neuron-specific viral vector [31]. Moreover, Kim *et al.* 2011 have shown recently that COS-7 cells transfected with α-synuclein, treated with MG-132 for 18 h had SUMO-1 staining of perinuclear α-synuclein inclusion bodies and SUMO-1 granules clustered nearby [27]. When subjected to anti-α-synuclein, IP SUMO-1-positive bands were detected in the transfected COS-7 cell extracts and blocking SUMOylation did not inhibit ubiquitination. Interestingly, SUMO-2 has also been implicated in the regulation of exosome mediated α-synuclein secretion, which may have relevance to the proposed prion model of disease spread in α-synuclein disease [66]. Although direct SUMOylation of α-synuclein was not detected in IP of purified pathological aggregates [24], this does not necessarily contradict the cell culture model studies, as SUMO modification would be expected to confer resistance to aggregation.

### 2.4. Chaperones, Co-Chaperones and Adaptor Proteins

Several chaperone and co-chaperone proteins, such as Hsc70, Hsp70, Hsp90 and Hsp40, play important roles in the protein degradative and protein misfolding/ aggregation response pathways, as mentioned previously, several of which interact with the SUMOylation cascade. Indeed, Balch and co-workers recently proposed the existence of the chaperone-rich quinary- or Q-body cytoplasmic compartment to participate in the decision between degradation or refolding (Figure 4; [67]). At the substrate level, several adaptor proteins are found to function in both UPS and autophagy pathways, where some proteins decide the catabolic route to be taken by the misfolded/aggregated protein. For instance, Parkin and VCP mediate both UPS and aggresome formation, while the BAG1/BAG3 ratio and CHIP select between autophagic and proteasomal degradation [34,44].

#### 2.4.1. Hsp90

Hsp90 is a multifunctional protective chaperone protein that is involved in the folding, re-folding and disaggregation of a diverse range of proteins as mentioned above. Indeed, Hsp90 inhibition can reduce α-synuclein aggregates by inducing macroautophagy [68]. Other important functions of Hsp90 include the maintenance of tertiary structure and ATP-ase activity of the proteasome, activation of protein kinases, activation of heat shock factor, *etc.* This functional diversity and specificity of Hsp90 is made possible by its conformational flexibility and dynamic association with various co-chaperone complexes. Wong and co-workers have shown recently that Hsp90 co-localized with SUMO-1 in α-synucleinopathy and tauopathy diseases and cell culture models [24]. Moreover, SUMOylation of Hsp90 at the *N*-terminus has been shown to promote recruitment of the Aha1 co-chaperone [23]. Furthermore, a screening study has identified Cdc37, a Hsp90 co-chaperone that is important for some autophagy pathways, is constitutively SUMOylated *in vivo* [69]. Thus, SUMOylation of lysosomal Hsp90, residing in the lysosomal lumen, may be important for its function in chaperone-mediated autophagy.

#### 2.4.2. HDAC6

At the regulatory level, inhibition or impairment of UPS stimulates autophagy via HDAC6-dependent mechanisms [61,70]. HDAC6 has a dual role in autophagy in aggresome formation [56,57] and autophagosome maturation [58] and hence this protein is essential for the successful completion of the autophagic process. HDAC6 is also important for mitophagy [71,72] and regulation of the ATP-dependent activity of Hsp90 [73]. Given that, SUMOylation can modulate the activities of other histone deacetylases like HDAC1 [74] and HDAC2 [75], it could possibly regulate HDAC6 activity in a similar manner. In fact, an *in vitro* assay has revealed HDAC6 SUMOylation by SUMO-1 and SUMO-2 [76]. Furthermore, SUMO-dependent recruitment of HDACs by specific proteins has also been demonstrated [74,75,77,78], although the mechanisms of this molecular specificity remain unclear. SUMO-dependent recruitment of HDAC6 is so far reported only by p300 protein [77]. Pharmacologic HDAC inhibitors induce hyperacetylation of Hsp90 and dissociate client proteins from the chaperone, leading to their degradation by the ubiquitin-dependent proteasomal pathway [77,79]. These studies highlight the fact that both general inhibition of HDACs and specific knockdown of HDAC6 can alter cytoplasmic-based processes [80,81]. Moreover, HDAC6 is found to accumulate in both Lewy bodies and α-synuclein glial cytoplasmic inclusions [82].

#### 2.4.3. VCP

VCP (P97) is a ubiquitin-selective chaperone that functions to disassemble protein complexes. Thus, shuttling factors of the UPS deliver client proteins to VCP for unfolding prior to delivering them to the proteasome [83]. Recently, it has been proposed that a balance between HDAC6 and VCP determines the fate of ubiquitinated proteins because VCP promotes proteasome-mediated protein degradation while HDAC6 stimulates aggresome formation via accumulating ubiquitinated proteins [84]. Nevertheless, aggresome formation was found to be impaired in VCP mutant cells and rescued by the expression of HDAC6 [85]. This suggests an interaction between HDAC6 and VCP is necessary for proper aggresome formation. Furthermore, VCP has been found to play a role in autophagosome maturation, which was first identified in mammalian cells. Mutants of p97 exhibit an upregulation of the autophagosomal markers, p62 and LC3II. Upon nutrient starvation-induced macroautophagy, cells expressing decreased p97 fail to degrade LC3II, demonstrating that p97 activity is required for the fusion of the autophagosome-lysosome, the formation of the autophagolysosome and eventually protein degradation [86]. Furthermore, VCP binds to SUMOylated targets and has been speculated to disassemble SUMO-stabilized protein assemblies, playing a role in SUMO-targeted ubiquitin-mediated degradation by the UPS [87].

#### 2.4.4. Parkin

Recent studies by Um *et al.* [88] have shown that the PD-linked E3 ligase, Parkin, that mediates K-63 ubiquitination associated with the aggresome pathway undergoes functional modulation through physical interaction with SUMO-1. Furthermore, Parkin-mediated K-63 ubiquitination was also found to be associated with NFκB signalling under cellular stress conditions, a pathway recently linked to non-selective macroautophagy of mitochonchondia (mitophagy) [89]. Moreover, Parkin-directed ubiquitination of mitochondrial surface proteins in concert with PINK1 and α-synuclein has been shown to target damaged mitochondria for macroautophagy [90,91]. Indeed, ubiquitin-like modifiers may play a more general role in mediating interactions between cargo and selective macroautophagy receptors, such as p62, and engaging the autophagosome nucleation machinery [92].

#### 2.4.5. TRAF6

Tumor necrosis factor-receptor associated factor 6 (TRAF6) is a RING-finger protein that acts as an E3 ubiquitin ligase. It is a common player in genetic and sporadic PD and a potential coordinator of the activation of autophagy and UPS [93]. TRAF6 binds misfolded mutant DJ-1 and α-synuclein. Interestingly, rather than conventional K-63 assembly, TRAF6 promotes atypical ubiquitin linkage formation to both PD targets that share K-63-, K-27- and K-29- mediated ubiquitination. Importantly, TRAF6 stimulates the accumulation of insoluble and polyubiquitinated mutant DJ-1 into cytoplasmic aggregates. In human post-mortem brains of PD patients, TRAF6 protein colocalizes with α-synuclein in LBs [94]. TRAF6 is an adaptor/scaffold protein that mediates several important signalling pathways, including the tumor necrosis factor-R:NF-κB pathway, involved in immune surveillance and inflammation. TRAF6 resides not only in the cellular cytoplasm but is also found in the nuclei of both normal and malignant B lymphocytes. TRAF6 does not possess a nuclear localization signal but enters the nucleus through the nuclear pore complex containing RanGap1. Nuclear TRAF6 is modified by SUMO-1, interacts with HDAC 1, and represses c-Myb-mediated transactivation [95]. Of the SUMO proteins, only SUMO-1 contains a nuclear localization signal that functions by transporting the cargo protein into the nucleus. Interestingly, SUMOylation of β-arrestin 2 attenuates binding to TRAF6, enhancing TRAF6 oligomerization and autoubiquitination [96]. The adaptor protein p62 is also found to contain a binding site for TRAF6 [34].

#### 2.4.6. CHIP

Hsp90 and the co-chaperone/ubiquitin ligase carboxyl terminus of Hsc70-interacting protein (CHIP) control the turnover of client proteins. CHIP is a RING-finger protein that acts as an E3 ubiquitin ligase and directs K-11 polyubiquitination. Upon complexation with BAG1 and Hsc70, CHIP polyubiquitinates BAG1, leading to proteasomal degradation (Figure 4) [97]. The ubiquitin ligase activity of CHIP resides in its U-box domain, while its interaction with the molecular chaperones Hsc70-Hsp70 and Hsp90 is via its TPR domain. A recent study provides evidence that the phosphorylation of the C-terminal region of both Hsp70 and Hsp90 is critical for the decision between folding and degradation of substrate proteins by the alteration of CHIP [98]. Thus, CHIP can act as a biomolecular switch between the folding-refolding and proteolytic pathways. In fact, a similar role for CHIP is observed in Aβ metabolism [99]. In addition, by ubiquitination of tau, CHIP appears to ameliorate the formation of tau neurofibrillary tangles associated with Alzheimer’s disease pathology [100]. A recent study reports redox regulation of the SUMO2/3-specific de-SUMOylating protease (SENP3) by CHIP via cross-interaction with Hsp90 [101]. Thus, upon mild oxidative stress, Senp3 is protected from CHIP-mediated ubiquitination and degradation, which results in elevated levels of Senp3. Although a similar mechanism is yet to be delineated for SUMO-1, the overall SUMOylation level in cells or SUMOylation of specific target proteins is likely modulated by the complex interplay of SUMOylating and deSUMOylating enzymes and accessory factors.

#### 2.4.7. P53 and ARF

Both p53 and ARF proteins that work cooperatively in their tumour-supression function have recently been reported to play roles in the induction of macroautophagy [102]. ARF (alternative reading frame) protein is generated by alternate splicing from the INK4 locus and is capable of both SUMOylation and K-63 polyubiquitination. ARF is reported to trigger SUMOylation of MDM2 and nucleophosmin [103], whilst nucleophosmin may sequester ARF in the nucleolus, preventing it from inhibiting MDM2. Studies have shown that the SENP3 and ARF are co-antagonistic in regulating the SUMOylation of nucleophosmin [104]. ARF also promotes K-63 polyubiqutination of COMMD1 and thereby directs it for proteasomal degradation [105]. ARF overexpression has also been shown to induce SUMOylation in human colon cancer cells. Furthermore, ARF showed specific interactions with the E2 SUMO ligase (Ubc9) making connections with target proteins indicating that ARF may act as an effective E3 SUMO ligase [106] and ARF-mediated induction of macroautophagy was dependent on cytoplasmic Hsp70 binding [107]. Interestingly, PUMA-independent macroautophagy induction by p53 is dependent on PIASy-mediated SUMOylation that also promotes increased p53 cytoplasmic localization [108].

## 3. Therapeutic Implications of SUMO Modulation of Selective Autophagy for Neuroprotection

SUMO-1 impinges on a number of different pathways related to protein folding/misfolding, the ubiquitin-proteasome system and the autophagy-lysosome system that could modify the toxicity of α-synuclein, either by direct SUMOylation of α-synuclein or indirectly by SUMOylation of factors, such as Hsp90. Figure 4 summarises the various pathways implicated for SUMO modification in the response to α-synuclein aggregates. We know that cross-talk between pathways enables selective and efficient degradation of specific proteins and organelles, and thereby enhances the ability of cells to cope with starvation, stress and pathological conditions. Hence, potentially the most effective therapeutic approach to alleviate the toxic burden caused by α-synuclein misfolding and/or aggregation would be to promote proteolysis in an efficient, yet specific manner. Of the different cellular mechanisms to eliminate toxic α-synuclein aggregates, the best route for therapeutic intervention would likely be the selective macroautophagy pathway, as pathologic aggregates are known to impair or inhibit both the UPS [109] and CMA [41] pathways due to the prerequisite unfolding step prior to degradation. Indeed, recent studies suggest that dysregulation of CMA occurs before substantial α-synuclein aggregation in PD [110]. Given the interplay between SUMOylation and ubiquitination and the specific effects they exert on both neurodegenerative disease and autophagy-lysosome pathway proteins, SUMOylation could be specifically modulated to influence the functions of multiple proteins and produce a protective outcome. It will be interesting to determine, for example, if inhibition of SUMOylation could inhibit the early chaperone-mediated autophagy response and if this might reciprocally upregulate macroautophagy and even modulate α-synuclein toxicity.

## 4. Conclusions

It is clear that SUMO-1 can modulate a number of key pathways influencing the formation and clearance of potentially cytotoxic α-synuclein species. Further studies are required to determine whether direct SUMOylation of α-synuclein or SUMOylation of other proteins is the dominant mechanism affecting the pathogenesis of α-synucleinopathy.
